# Dissipated power and induced velocity fields data of a micro single dielectric barrier discharge plasma actuator for active flow control^☆^

**DOI:** 10.1016/j.dib.2015.08.011

**Published:** 2015-08-28

**Authors:** E. Pescini, D.S. Martínez, M.G. De Giorgi, L. Francioso, A. Ficarella

**Affiliations:** a University of Salento, Department of Engineering for Innovation, Via per Monteroni, 73100 Lecce, Italy; b Universidad Politécnica de Cartagena, Dep. Ingeníeria Térmica y de Fluidos, c/Dr. Fleming s/n, 30202 Cartagena, Murcia, Spain; c CNR-IMM, Istitute for Microelettronics and Microsystems, Via per Monteroni, 73100 Lecce, Italy

**Keywords:** Micro SDBD, Plasma induced velocity, PIV measurements, DBD CFD modeling, Wall jet

## Abstract

In recent years, single dielectric barrier discharge (SDBD) plasma actuators have gained great interest among all the active flow control devices typically employed in aerospace and turbomachinery applications [1,2]. Compared with the macro SDBDs, the micro single dielectric barrier discharge (MSDBD) actuators showed a higher efficiency in conversion of input electrical power to delivered mechanical power [3,4]. This article provides data regarding the performances of a MSDBD plasma actuator [5,6]. The power dissipation values [5] and the experimental and numerical induced velocity fields [6] are provided. The present data support and enrich the research article entitled “Optimization of micro single dielectric barrier discharge plasma actuator models based on experimental velocity and body force fields” by Pescini et al. [6].

A dedicated activity was devoted to microelectronic technology adoption for copper (Cu) electrode fabrication on glass–reinforced epoxy laminate (FR4) substrates, together with batch production of electrodes with photolithographic techniques. Actuation under sinusoidal voltage with amplitude up to 7 kV and frequency up to 2.5 kHz was considered. The working fluid was air, initially quiescent.

Electrical characterization was done by measuring the voltage–current characteristic curves. The experimental velocity data were retrieved by Particle Image Velocimetry (PIV), while the numerical ones were obtained by a Computational Fluid Dynamic (CFD) model implemented in OpenFOAM, validated with the above mentioned experimental values.

The data provided here are suitable for comparing the developed micro actuator with others characterized by different geometry and/or constructive materials. Moreover, they can be also employed for validations of other CFD codes, which aim to predict the plasma actuation effect on the flow.

**Specifications table**

**Table:** 

Subject area	Aerospace engineering and fluid dynamics.
More specific subject area	Active flow control, CFD modeling.
Type of data	Power dissipation data (table)
Velocity data files (dat format).
How data was acquired	Experimental power dissipation: by recording the voltage–current curves with an oscilloscope (Picoscope 5204) and processing with Matlab.
Experimental velocities: by two-dimensional (2D) PIV measurements (Dantec system).
Numerical velocities: by 2D CFD model implemented in OpenFOAM.
Data format	Experimental power dissipation: average power dissipation calculated from the recorded voltage–current curves.
Experimental velocities: Cartesian coordinates and components of the average velocity fields, obtained from the processed images.
Numerical velocities: raw velocity fields and corresponding mesh grid coordinates.
Experimental factors	The micro-single dielectric barrier discharge plasma actuator was built by optical lithography fabrication method, allowing a fine control of the electrode gap and the dimensions with high manufacturing reliability. The electrode material was Cu and the dielectric material was FR4 dielectric layer.
Experimental features	The working fluid was air initially quiescent. The laboratory temperature was 25 °C and the pressure was 1.01325 bar. To reduce the effect of any external disturbance on the measured velocity, the tests were conducted in a plexiglas closed box.
Data source location	University of Salento – Lecce, Italy.
Data accessibility	The power dissipation data are directly provided with this article. Velocity data are provided in supplementary files directly with this article.

**Value of the data**

• Many works in the literature are related to macro SDBD plasma actuators; the present data are related to the dissipated power and the flow field induced by a MSDBD actuator.
• The actuator efficiency in conversion of input electrical power to delivered mechanical power is increased by the adoption of MSDBDs.
• The actuation effect of MSDBD plasma actuators is still not well modeled in the literature. The CFD data here provided a good prediction of the micro actuation effect.
• The provided data are suitable for comparing the developed micro actuator with others characterized by different geometry and/or constructive materials.
• The experimental and numerical velocity data can be used to calibrate/validate other numerical models, which aim to predict the plasma actuation effect on the flow.

## 1 Data

During the MSDBD operation an electrohydrodynamic (EHD) plasma-induced body force acted on the surrounding ambient (neutrally charged) air, drawing it towards the actuator surface and expelling it away from the exposed electrode. By increasing the applied voltage frequency or amplitude, the actuator power dissipation increased but also the plasma induced velocity in the area downstream of the exposed electrode (downstream area) was enhanced. Such area was experimentally investigated. Experimental velocity measurements were started once the flow was fully established in the entire measurement box (at about 10 s after the starting of the actuation [6]). Steady simulations were carried out and validated by the experimental velocity data. The numerical data set complemented and extended the experimentally retrieved velocity field. All data are thus available for calculating the fluid mechanic power and the provision of the power dissipation data are useful for evaluating the efficiency of the investigated device.

## 2 Experimental design, materials and methods

The investigated MSDBD was manufactured by photolithographic technique and allocated in a groove made in the middle of a Plexiglas square flat plate. The electrodes material was Cu; they were separated by a FR4 dielectric layer and patterned along the streamwise (*x*) direction by a gap.

The working fluid was air, initially quiescent. To reduce the effect of any external disturbance on the velocity measurements, the tests were conducted in a Plexiglas closed box, having the base coincided with the flat plate and a height equal to 250 mm. The laboratory temperature was 25 °C and the pressure was 1.01325 bar.

**Fig. 1:**
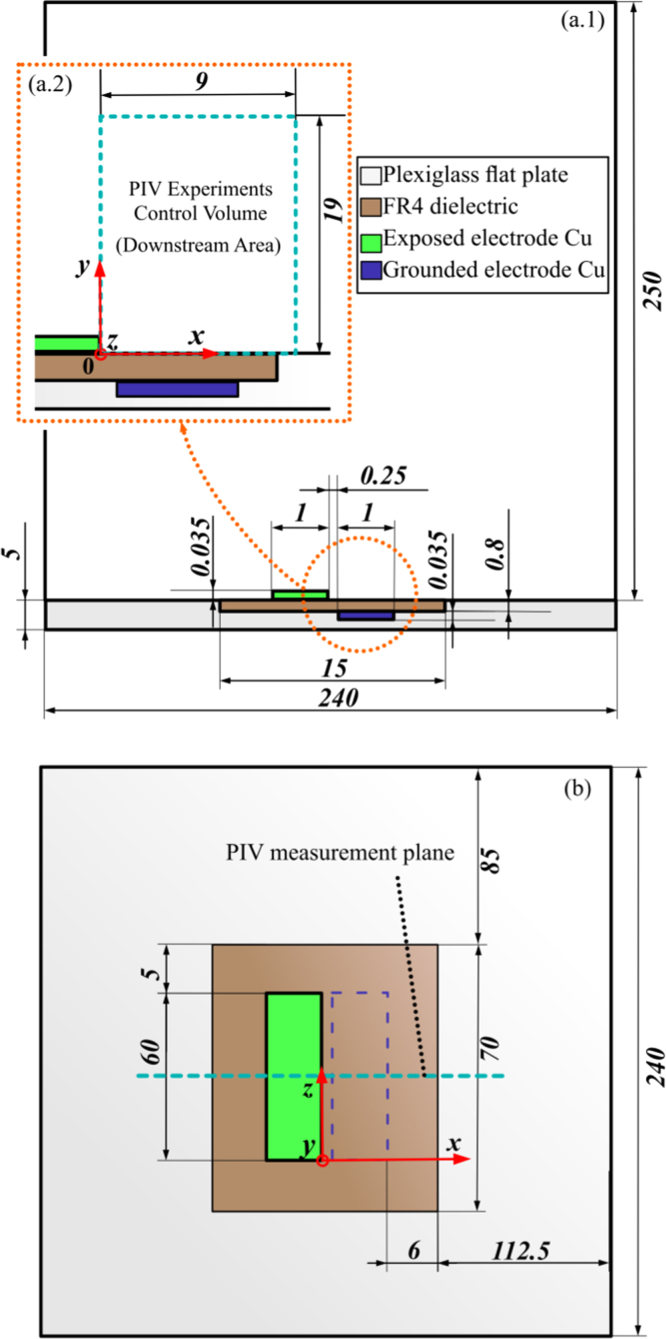
Cross-sectional schematic of the tested MSDBD, flat plate where it is located and bounding box (dimensions are expressed in mm): (a.1) side view, (a.2) detail of the PIV domain and reference system, (b) top view with indication of the PIV measurement plane located at the midspan section of the actuator.

A cross-sectional schematic of the flat plate with the tested MSDBD allocated and of the bounding box is reported in Fig. 1.

**Fig. 2:**
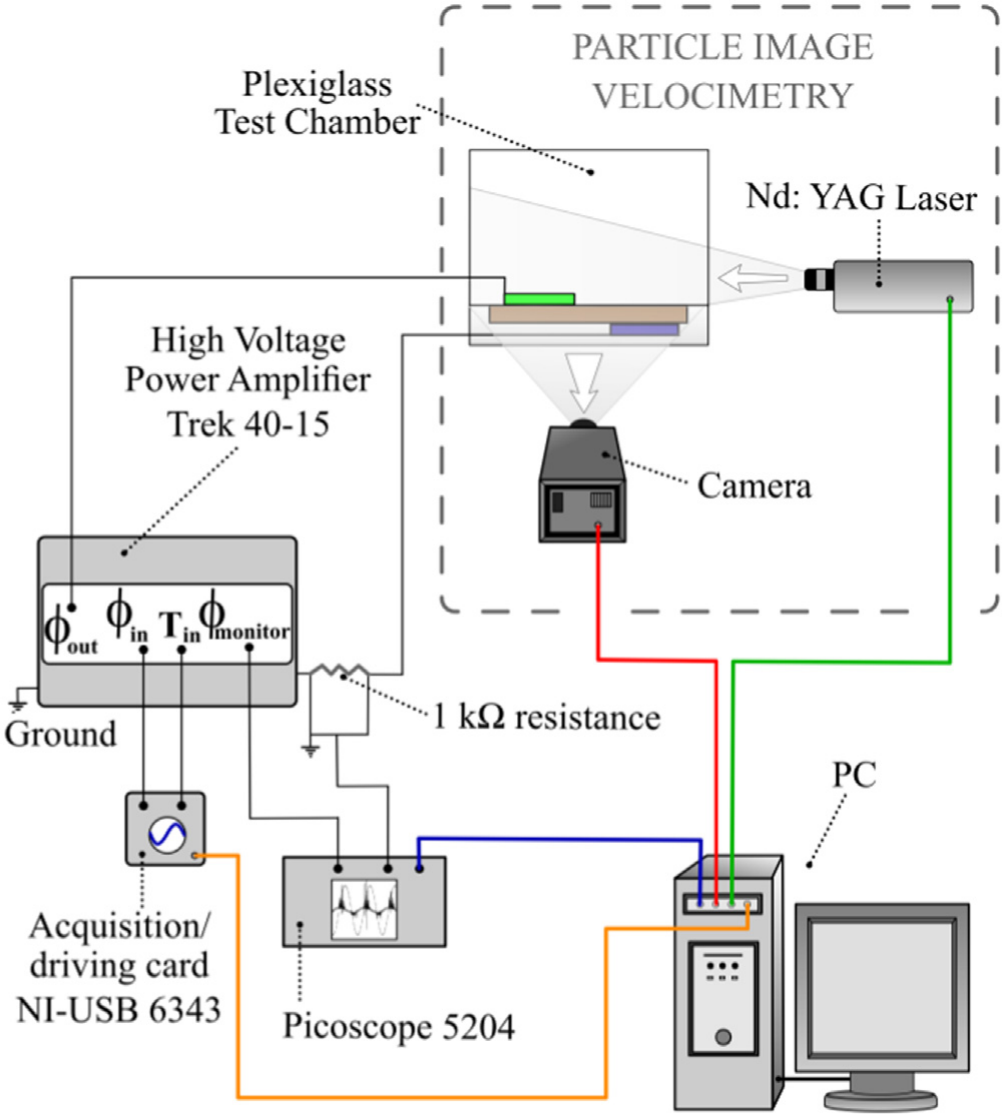
Schematic view of the experimental setup and instrumentation.

The experimental setup consisted of two separate systems, electrical and PIV, for the simultaneous measurements of the MSDBD voltage–current curves and the induced velocity field. A sketch of the experimental arrangement is reported in Fig. 2.

The electrical system was composed by a dedicated PC and an acquisition/driving card (*NI-USB 6343*), which triggered and supplied a high voltage (HV) amplifier (*Trek 40-15*) with a sinusoidal voltage waveform *ϕ*_i*n*_. The actuator׳s upper electrode (denoted by exposed electrode in Fig. 1) was exposed to the surrounding air, connected to the output *ϕ*_*out*_ of the HV amplifier and supplied with sinusoidal HV characterized by zero DC offset, amplitude ϕ^ values ranging from 5 to 7 kV (respectively from 10 kV to 14 kV peak to peak) and frequency f^ values ranging from 1 to 2.5 kHz. The lower electrode (denoted by grounded electrode in Fig. 1) was instead grounded, embedded between the FR4 and the Plexiglas. A sampling resistance of 1000 Ω was placed in series between the actuator and the ground. Both the amplifier voltage output monitor *ϕ*_*monitor*_ and the resistor terminals were connected to an oscilloscope (*Picoscope 5204)* and the respective signals were recorded with and accuracy of ±3%, at a sampling rate of 31.25 MHz. The average electric power dissipation $\overset{¯}{P_{el}}$was calculated by averaging the instantaneous electrical power dissipation over 33 actuation periods, sufficient for getting a good accuracy in the data [7]. Numerical integration was performed by the trapezoidal method. The error in the $\overset{¯}{P_{el}}$ estimation was estimated by standard uncertainty analysis methodology [8] and resulted in a percent error of ±4.24%.

The different test cases [6] are summarized in Table 1, together with the respective $\overset{¯}{P_{el}}$ data.

The PIV system consisted of a dedicated PC, a Nd:YAG dual cavity pulsed 532 nm laser (*NANO-L 200-15*) and a FlowSense EO 4M camera (2048×2048 pixels). A 2D PIV configuration was chosen for characterizing the plasma induced flow, as the large span of the actuator ensured minimal 3D effects [9]. A light sheet ≈1 mm thick was generated at the midspan section of the actuator (see Fig. 1(b)) and 200 pairs of PIV instantaneous images were acquired at 6 Hz. Incense smoke seeding was used; the Stokes number *St* was also evaluated and it resulted of the order 10^−4^ for all the tested conditions, meaning a good fluidic response from the tracer particles [5]. The investigated PIV domain and the adopted reference system are shown in Fig. 1(a.2). More details about the PIV setup are reported in [5,6].

The PIV images post-processing was performed by using the Dantec Dynamic Studio v3.40 software. Details about the digital analysis and the adopted correlation method are reported in Pescini et al. [5–7]. The spatial resolution of the processed PIV data was 0.2 mm/pixel. The mean velocity fields, provided with this data article were obtained by averaging the instantaneous PIV velocity maps of obtained valid vectors. The accuracy for instantaneous velocity measurements was estimated to be around 1%, based on correlation peak estimation error of 0.1 pixels (provided by Dantec Dynamics [10]) and maximum particle displacement around 7 pixels [7]. The highest statistical percent error on the maximum velocity magnitude was ±3.16% [5,6]. The error on the measured *x* and *y* position, estimated by the guidelines reported in [11], was about ±0.14 mm.

The experimental *x* and *y* mean velocity fields, together with their respective statistical percent error (calculated as reported in [7]) and the cartesian coordinates, are provided as “dat” files for all the tested conditions reported in Table 1. The adopted reference system is the one reported in Fig. 1(a.2).

## 3 Numerical approach

Among the three different models tested in [6], the dual potential model (DPM) data are reported here, being the ones that provided the best agreement with the experimental values. The plasma induced body force was modeled by the DPM of Suzen et al. [12], and its averaged value was implemented in the steady Navier Stokes equations as a body force term.

The effect in the flow of the force oscillations was evaluated in [6]. It was found that the oscillatory effect of the force can be ignored and only the mean value of the plasma induced force affects the flow: the problem could be then assumed steady. Therefore, the induction of the flow in this problem is different from other mechanisms where boundary oscillations drive the fluid mass flux [13].

A 2D numerical domain was used, whose dimensions corresponded to the ones in the experimental cross sectional view reported in Fig. 1(a.1). According to the Reynolds number retrieved by the experimental data [6], laminar simulations were performed.

The model was developed in OpenFOAM, an open-source software based on the finite volume method. A 2D, structured non-uniform mesh of hexahedra elements was created in order to accurately control the size and number of cells in the domain. The grid was refined towards the electrodes and the dielectric surface in order to reduce the computational cost, reaching the grid independence. The total number of cells was of 209,800, where 119,650 were on the fluid mesh. A second order discretization scheme was used. Its accuracy, estimated according to the method proposed in [14] resulted in ±0.01%.

The numerical *x* and y velocity fields, together with their respective mesh grid coordinates, are provided as *“dat”* files for all the tested conditions reported in Table 1. The adopted reference system is the one reported in Fig. 1(a.2).

## Figures and Tables

**Table 1:** Test cases and respective actuator dissipated power.

Test case	ϕ^kV	f^kHz	$\overset{¯}{P_{el}}\left(W\right)$

A	5	1	0.2018
B	7	1	0.7480
C	5	2.5	0.5403
